# Position of draining venous cannula in extracorporeal membrane oxygenation for respiratory and respiratory/circulatory support in adult patients

**DOI:** 10.1186/s13054-018-2083-0

**Published:** 2018-06-15

**Authors:** B. Frenckner, M. Broman, M. Broomé

**Affiliations:** ECMO Centre Karolinska, Karolinska University Hospital, Solna, 171 76 Stockholm, Sweden

**Keywords:** Veno-venous ECMO, Veno-arterial ECMO, Draining cannula, Flow direction, Recirculation, North-south syndrome, Differential hypoxemia, Dual circulation

## Abstract

Extracorporeal membrane oxygenation (ECMO) is used in critically ill patients with severe pulmonary and/or cardiac failure. Blood is drained from the venous system and pumped through a membrane oxygenator where it is oxygenated. For pulmonary support, the blood is returned to the patient via a vein (veno-venous ECMO) and for pulmonary/circulatory support it is returned via an artery (veno-arterial ECMO).

Veno-venous ECMO can be performed either with a single dual-lumen cannula or with two separate single-lumen cannulas. If the latter is chosen, flow direction can either be from the inferior caval vein (IVC) to the right atrium or the opposite. Earlier research has shown that drainage from the IVC yields less recirculation and therefore the IVC to right atrium route has become the standard in most centers for veno-venous ECMO with two cannulas. However, recent research has shown that recirculation can be minimized using a multistage draining cannula in the optimal position inserted via the right internal jugular vein and with blood return to the femoral vein. The clinical results with this route are excellent.

In veno-arterial ECMO the most common site for blood infusion is the femoral artery. If venous blood is drained from the IVC, the patient is at risk of developing a dual circulation (Harlequin syndrome, North-South syndrome, differential oxygenation) meaning a poor oxygenation of the upper part of the body, while the lower part has excellent oxygenation. By instead draining from the superior caval vein (SVC) via a multistage cannula inserted in the right internal jugular vein this risk is neutralized.

In conclusion, the authors argue that draining blood from the SVC and right atrium via a multistage cannula inserted in the right internal jugular vein is equal or better than IVC drainage both in veno-venous two cannula ECMO and in veno-arterial ECMO with blood return to the femoral artery.

## Background

Extracorporeal membrane oxygenation (ECMO) is used in critically ill patients with severe pulmonary and/or cardiac failure. Blood is drained from the venous system and pumped through a membrane oxygenator, where it is oxygenated and decarboxylated. For pulmonary support, the blood is returned to the patient via a vein (veno-venous (V-V) ECMO) and for pulmonary and/or circulatory support it is returned via an artery (veno-arterial (V-A) ECMO). As oxygen is predominantly transported bound to hemoglobin (Hb), the oxygen delivery (D_ecmoO2_ in mL O_2_/min) from the ECMO circuit can easily be calculated from the simplified formula as D_ecmo_O_2_ = Flow × [Hb] × 0.0134 × (100 – Sat_preox_), where Flow is the ECMO pump flow (l/min), [Hb] is hemoglobin concentration (g/L), and Sat_preox_ is the preoxygenator saturation (%). A high flow rate and low Sat_preox_ will result in a high oxygen delivery. ECMO is used in all age groups, and several randomized controlled trials have shown that it is lifesaving both in neonates [1–3] and in adult patients [4].

To fully support the oxygenation of a patient, ECMO flow needs to be in the range of the normal cardiac output. As the flow resistance in tubes is inversely proportional to the fourth power of the radius (Poiseuille’s Law), the diameter of the cannulas is of great importance and large cannulas require large vessels. Central cannulation (i.e., direct cannulation of the right atrium and aorta) requires a sternotomy and is mostly used postcardiotomy. Peripheral cannulation outside the thorax and abdomen is less invasive and is the normal route for most ECMO patients. The largest available vessels, and those used for ECMO in adults and older children, are the internal jugular vein (IJV), the femoral vein, the subclavian artery, and the femoral artery.

### Veno-venous ECMO

**Fig. 1:**
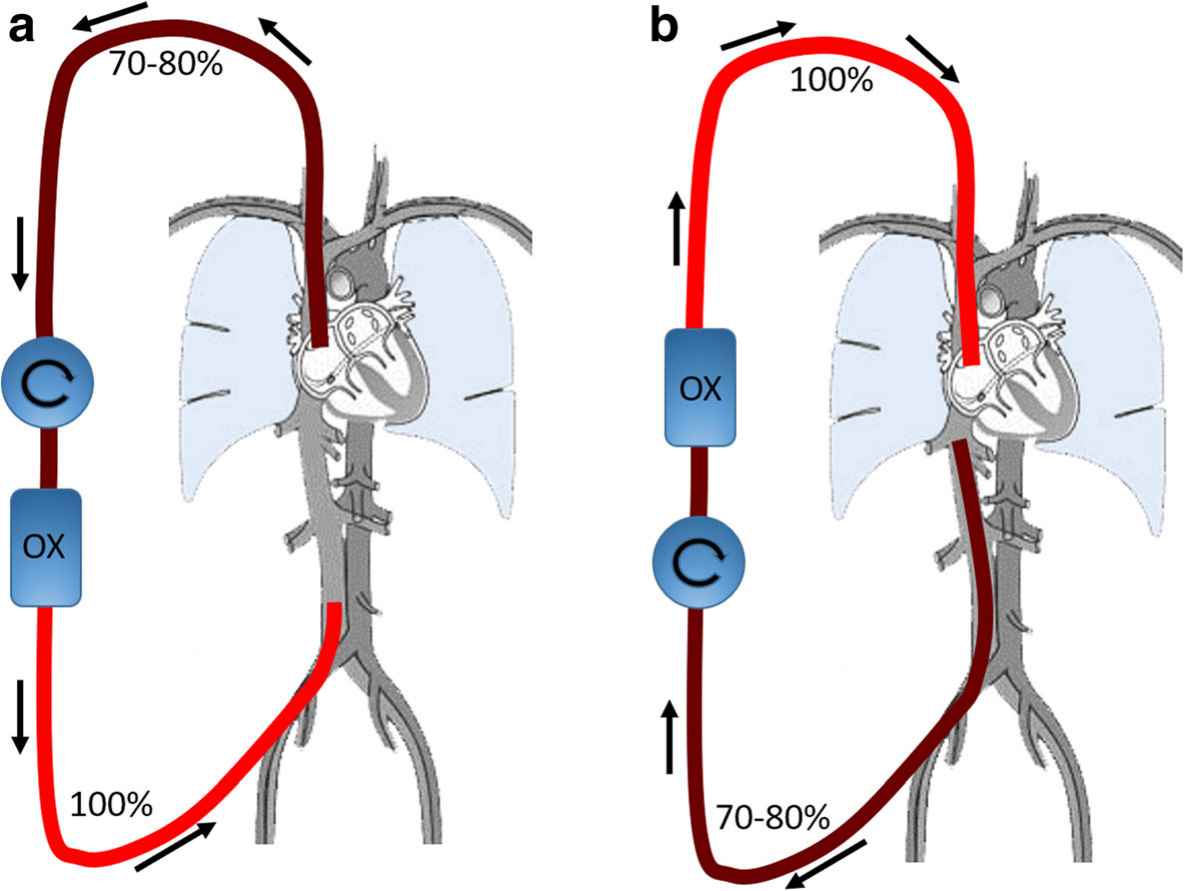
Schematic drawing of the circuit in V-V ECMO. **a** Draining from the SVC/RA and infusing into the femoral vein; **b** draining from the IVC and infusing into the SVC. Preoxygenator saturation can be expected to be 70–80% depending on the amount of recirculation. OX oxygenator

In V-V ECMO, the oxygenated blood is returned into the venous system. Some of this blood will in turn be drained by the draining cannula, which is referred to as recirculation (Rf). A high Rf will raise the Sat_preox_ and therefore lower the oxygen delivery. To support the patient efficiently, Rf should thus be as small as possible. Rf is affected by many factors including positioning of the cannulas, ECMO flow, ECMO flow direction, cardiac output, and intrathoracic/intra-abdominal pressures [5]. V-V ECMO can be accomplished either through a dual-lumen cannula (with one draining canal and one infusion canal) or through two single-lumen cannulas. When the latter mode is used, possible configurations are femoral vein to IJV, IJV vein to femoral vein, and femoral vein (one side) to femoral vein (other side). One should also bear in mind that it is not the insertion site that matters, but *from where the blood is drained and reinfused*. Draining cannulas are equipped with an end-hole and several side-holes. So-called multistage cannulas have side-holes at several different levels and, if these lie freely and unobstructed in a vessel, they will primarily drain from the most proximal side-holes (i.e., from the side-holes furthest away from the tip). The configurations are therefore better described as IVC–RA (drainage from the inferior caval vein (IVC) through a femoral cannula and return to the right atrium (RA) via a cannula in the right IJV or in the other femoral vein) or RA–IVC (drainage from the RA and infusion in the IVC) (Fig. 1). With a multistage cannula inserted in the jugular vein, blood will be drained both from the superior caval vein (SVC) and the RA (i.e., SVC/RA). In the early ECMO era the RA–IVC route was traditionally routine in the United States [6], while the IVC–RA route was used in Europe [7]. Rich et al. [8] prospectively compared efficient oxygenation between RA–IVC and IVC–RA flow directions and concluded that recirculation was less when draining from the IVC and returning blood via the RA. Furthermore, they were able to maintain a higher ECMO flow when using this flow direction. Following this, the policy was changed in North America and today the majority of ECMO centers use the IVC–RA route for two single-lumen cannula V-V ECMO [9]. However, in the study of Rich et al. the patients were all paralyzed and subjected to inverse-ratio pressure-controlled ventilation. As pointed out by the authors, this may have contributed to a lesser ability to drain from the RA and improvement in the ability to drain from the IVC. Furthermore, the study was performed during the first day of support and no attempt had been made to withdraw fluid. The authors mention that intravascular volume depletion may compromise IVC–RA flow more than RA–IVC flow, thereby limiting the value of their finding. In a recent study, the amount of recirculation was studied in RA–IVC ECMO patients by an ultrasound dilution technique [10]. The authors found, that by using a multistage cannula (Maquet HLS, 25 French/38 cm, Maquet Cardiopulmonary/Getinge Group, Hirrlingen, Germany) inserted via the right IJV with the tip in the RA as compared with a conventional lighthouse tip cannula (Bio-Medicus 23 French/25 cm, Medtronic Europe Sárl, Tolochenaz, Switzerland) inserted in the same way, Rf was reduced from an average of 38% to 19%. This multistage cannula has side-holes up to 10 cm from the tip and, in adult patients, it can be inserted in the right jugular vein without any risk of proximal side-holes being too close to the skin. The lower Rf was explained by a larger proportion of SVC drainage via the proximal side holes. In Stockholm, we have a tradition of performing V-V ECMO via the SVC/RA–IVC route and have demonstrated excellent results [11]. By using a multistage cannula in the SVC with exact placement of its tip at the entry of the RA, recirculation is minimized.

### Veno-arterial ECMO

**Fig. 2:**
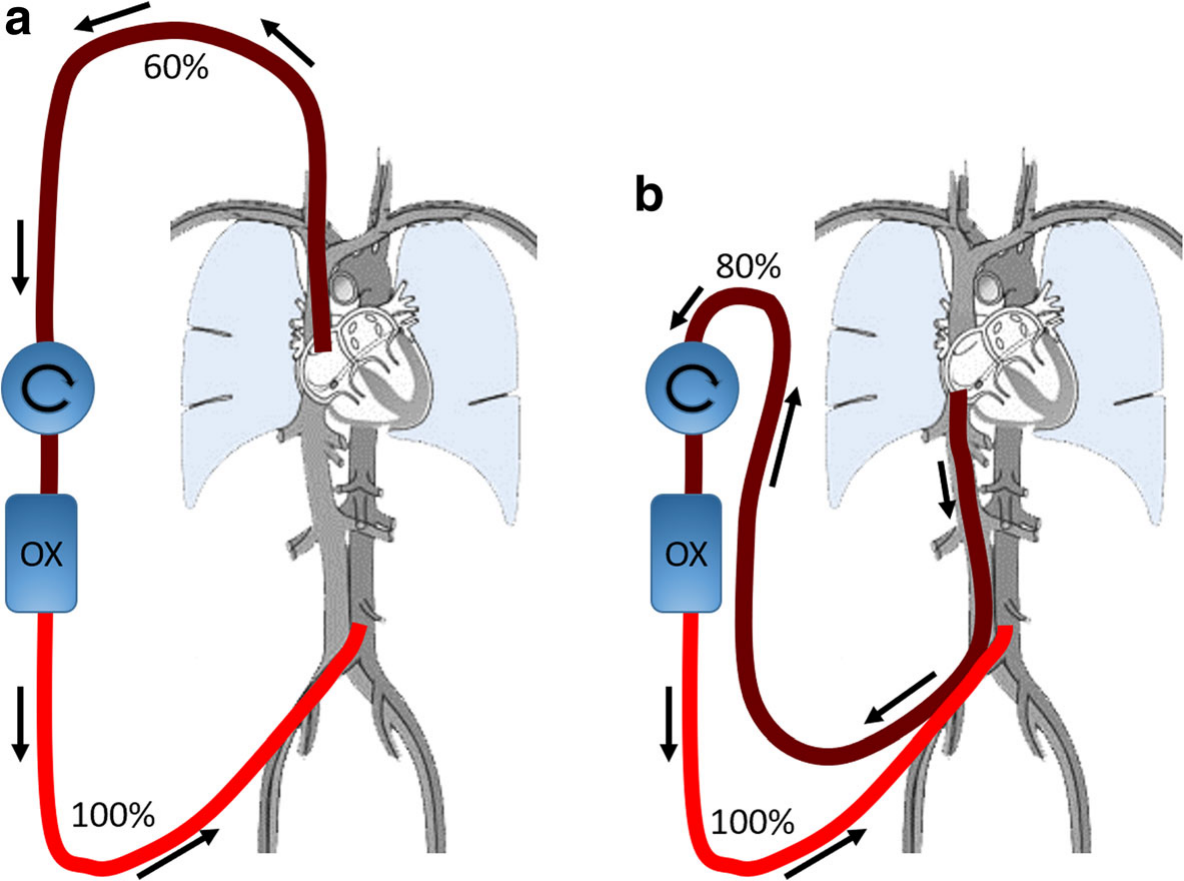
Schematic drawing of the circuit in V-A ECMO. **a** Draining from the SVC/RA and infusing into either femoral artery; **b** draining from the IVC and also infusing into either femoral artery. The patient has an ejecting heart and severely failing lungs. **a** Illustrates the situation where the highly saturated IVC blood enters the pulmonary circulation and is further ejected from the left ventricle to the upper part of the body. The poorly saturated SVC blood is, in turn, drained back to the ECMO machine. **b** Illustrates instead the situation when the highly saturated IVC blood is drained back to the ECMO machine. In this scenario the ECMO circulation is poorly mixed with the patient’s native circulation resulting into a dual circulation situation. OX oxygenator

**Fig. 3:**
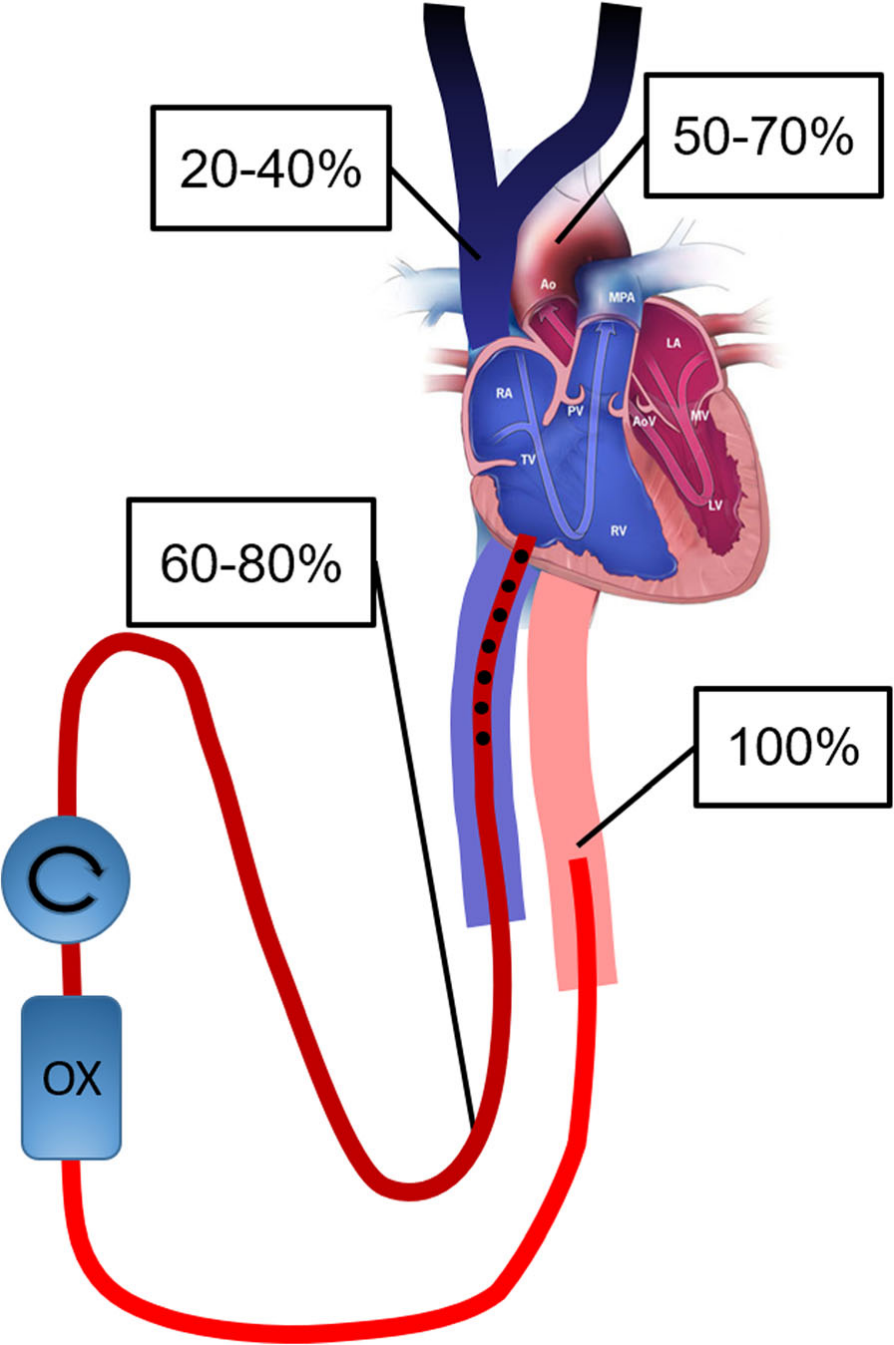
Schematic drawing exemplifying dual circulation in V-A ECMO with severe pulmonary failure when draining from the IVC and reinfusing into the femoral artery. Although preoxygenator saturation is acceptable at 60–80%, the arterial saturation in the aortic arch may be 50–70% and SVC saturation as low as 20–40%. The SVC desaturation may be unnoticed since the venous blood drained to the ECMO circuit mainly originates from the IVC, where venous saturations usually show high normal values. OX oxygenator

In V-A ECMO, blood is returned to a major artery. The most commonly used artery in adults is the femoral artery, meaning there will be a retrograde flow in the descending aorta meeting the blood that is ejected from the left ventricle (LV) into the ascending aorta (Fig. 2). The level of the mixing zone is dependent on the native cardiac output and the ECMO flow. In patients with pulmonary failure, the blood ejected from the LV is desaturated and will predominantly support the upper part of the body (coronary, subclavian, and carotid arteries). If the venous blood is drained from the IVC (i.e., via a femoral cannula) the patient is at risk of developing a dual circulation (Harlequin syndrome, North-South syndrome, differential oxygenation) meaning that the highly oxygenated blood from the ECMO machine enters the descending aorta, supporting the lower body (splanchnic circulation, kidneys, legs, etc.) and drains into the IVC and further to the ECMO circuit [12]. On the other hand, the desaturated blood ejected from the LV that supports the upper body (heart, brain, etc.) drains into the SVC and is pumped through the failing native lungs to the left atrium and LV without being mixed with the oxygenated blood from the ECMO machine. This results in low arterial and venous saturations in the upper body and a well oxygenated lower body (Fig. 3). Early assessment is accomplished by blood gas analysis showing that Sat_preox_ exceeds or is similar to saturation in the upper body. If instead blood is drained from the SVC there will be a favorable mixture of the circulations in the upper and lower body. The relatively well-saturated blood from the IVC enters the pulmonary circulation (instead of being drained to the ECMO machine) and is ejected from the LV to support the upper body. The most desaturated blood in this situation is in the SVC and is drained to the ECMO machine instead of entering the pulmonary circulation. Furthermore, by draining the most desaturated blood, more oxygen can be delivered to the patient. The efficiency of draining from the SVC to avoid dual circulation in V-A ECMO has been shown both experimentally, in a computerized model, and in clinical practice [13, 14] (Table 1). In patients on V-A bypass only for circulatory support, the blood ejected by the LV may be well oxygenated (if not pulmonary edema) and the venous drainage site is therefore often of less importance with regard to dual circulation. In patients with extremely low native cardiac output, the retrograde flow from the ECMO machine will sometimes reach the aortic arch, but in this situation flow stagnation in the LV, pulmonary congestion, and coronary hypoxemia may be threatening. However, in patients with circulatory support, pulmonary function may also deteriorate due to LV failure worsening due to ECMO-induced high afterload, atelectasis, infection, etc. The risk of dual circulation syndrome therefore always exists when blood is drained from the IVC. We therefore argue that in V-A ECMO with blood return in the femoral artery venous blood should routinely be drained from the SVC and upper part of the RA. In our opinion the best way to accomplish this is with a multistage cannula inserted via the right IJV and with its tip located at the entrance to the RA. An exception from the recommended route for drainage is when the patient is cannulated during refractory cardiac arrest. In such cases cannulation via the groin may be quicker especially as the neck may be difficult to reach due to the resuscitation.

**Table 1:** Upper body oxygenation during V-A ECMO for severe respiratory failure with infusion in one femoral artery

	Upper body oxygen saturation	
	Drainage from IVC	Drainage from SVC
Experimental study in sheep [13]	35%	75%
Patient case report [14]	57%	85%
Simulation model [14]	64%	81%

Blood was initially drained from the inferior caval vein (IVC) resulting in a low upper body oxygenation (dual circulation), but when the draining site was changed to the superior caval vein (SVC) a marked improvement occurred. Saturation was measured in the aortic root in the experimental study and in the right hand in the patient and in the simulation

Another common way to solve the oxygenation problem when/if the dual circulation phenomenon is discovered in V-A ECMO via femoral vessels is to implant an extra venous reinfusion cannula via the IJV. V-A ECMO is thereby converted to V-AV [15]. By doing this, oxygen is introduced to the upper (native) circuit increasing central venous oxygen content. The saturation of the blood leaving the LV will be higher compared with before. However, it should be noted that the Sat_preox_ most likely also increases due to recirculation (from oxygenated blood in the RA to the draining cannula in the IVC), and thus the total amount of oxygen it is possible to deliver via the ECMO support may not increase but rather decrease if there is a limit in compensatory maximum ECMO flow. Drainage capacity may be restricted by the physical properties of the draining cannula, or hypovolemia, etc. An alternative in V-A ECMO is to return the blood in the subclavian artery instead of the femoral artery. This would eliminate the risk for a dual circulation as the fully oxygenated blood from the ECMO machine will be equally distributed in the body. However, subclavian artery cannulation is technically more demanding than femoral artery cannulation and also carries the risk of hyperperfusion of the ipsilateral arm, surgical site bleeding [16], and cerebral embolization.

Although serious complications such as right ventricular rupture and cardiac tamponade have been described using double-lumen ECMO cannulas in the RA inserted via the IJV and with its tip located in the IVC [17], we consider having large single-lumen draining cannulas inserted via the right IJV in the upper part of the RA as a safe procedure. Since the start of our adult ECMO program in 1996 and until 2018 we have treated 462 adult patients on ECMO with such draining without any cardiac perforations.

## Conclusion

In conclusion, we argue that drainage from the RA and SVC via a multistage cannula inserted in the right IJV is an ideal alternative in ECMO for respiratory and respiratory/circulatory support. It enables an efficient drainage from the upper part of the body. In V-A ECMO with infusion in the femoral artery, dual circulation is avoided. In V-V ECMO, drainage from the RA/SVC yields excellent results with low recirculation within acceptable limits. Furthermore, if a patient on V-V ECMO needs to be converted to V-A ECMO, the ideal draining cannula is already in place.

## Abbreviations

D_ecmo_O_2_: Oxygen delivery from the ECMO circuit
ECMO: Extracorporeal membrane oxygenation
Hb: Hemoglobin
IJV: Internal jugular vein
IVC: Inferior caval vein
LV: Left ventricle
RA: Right atrium
Rf: Recirculation
Sat_preox_: Preoxygenator saturation
SVC: Superior caval vein
V-A: Veno-arterial
V-V: Veno-venous
